# Estimation of Salivary Glucose, Calcium, Phosphorus, Alkaline Phosphatase, and Immunoglobulin A among Diabetic and Nondiabetic Children: A Case-Control Study

**DOI:** 10.5005/jp-journals-10005-1488

**Published:** 2018-04-01

**Authors:** Kalyani Uppu, Suzan Sahana, Ghanashyam P Madu, Aron AK Vasa, Sowjanya Nalluri, Kumar J Raghavendra

**Affiliations:** 1 Postgraduate Student, Department of Pedodontics and Preventive Dentistry, Saint Joseph Dental College, Eluru, Andhra Pradesh, India; 2 Professor, Department of Pedodontics and Preventive Dentistry, Saint Joseph Dental College, Eluru, Andhra Pradesh, India; 3 Professor and Head, Department of Pedodontics and Preventive Dentistry, Saint Joseph Dental College, Eluru, Andhra Pradesh, India; 4 Professor, Department of Pedodontics and Preventive Dentistry, Saint Joseph Dental College, Eluru, Andhra Pradesh, India; 5 Professor, Department of Pedodontics and Preventive Dentistry, Saint Joseph Dental College, Eluru, Andhra Pradesh, India; 6 Senior Lecturer, Department of Pedodontics and Preventive Dentistry, Saint Joseph Dental College, Eluru, Andhra Pradesh, India

**Keywords:** Alkaline phosphatase, Calcium, Nondiabetic healthy children, Phosphorus, Salivary glucose, s-IgA, Type I diabetic children.

## Abstract

**Introduction:**

Saliva is vital for oral health and helps to maintain oral homeostasis. It may show qualitative and quantitative variations owing to any changes in the systemic health. Diabetes mellitus (DM) is a metabolic disease and the individuals may be at higher risk for oral health problems.

**Objective:**

The study was aimed to estimate the levels of various salivary components among diabetic and nondiabetic children with similar caries status and also to analyze possible association between caries status and possible caries determinants in the saliva of diabetic children.

**Materials and methods:**

A total of 70 children in the age group of 6 to 13 years with minimal dental caries (Decayed, Missing and Filled Teeth index (DMFT/dmft >1 and <5)) were selected. Group I comprised of type I diabetic children and on medication for diabetes and group II included healthy nondiabetic children. Salivary samples were collected from the participants by passive drool method and estimation of all salivary parameters was done using autoanalyzer.

**Results:**

Statistical analyses were done using Student’s t-test and results are presented as mean ± standard deviation (SD). There was a highly significant difference in mean glucose value between diabetic and nondiabetic children. Levels of salivary calcium, phosphorus, and salivary immunoglobulin A (s-IgA) did not show any significant difference between the two groups. There was also a statistically significant difference in the alkaline phosphatase (AP) levels, which was found to be higher in diabetics.

**Conclusion:**

An elevation in the levels of salivary glucose and AP was evident in diabetic children, which can be a risk marker for dental caries. There was no correlation in the levels of salivary calcium, phosphorus, and s-IgA levels among diabetic and healthy children.

**Clinical significance:**

The salivary factors evaluated in the study may prove to be useful measures of caries experience in diabetic children.

**How to cite this article:** Uppu K, Sahana S, Madu GP, Vasa AAK, Nalluri S, Raghavendra KJ. Estimation of Salivary Glucose, Calcium, Phosphorus, Alkaline Phosphatase, and Immunoglobulin A among Diabetic and Nondiabetic Children: A Case-Control Study. Int J Clin Pediatr Dent 2018;11(2):71-78.

## INTRODUCTION

Oral cavity is an extremely dynamic and unique environment, the only place in the body where mineralized tissues are exposed to external environment that involves complex interactions between different surfaces constantly exposed to saliva. To a large extent, saliva fosters oral health and no other etiological factor influences the outcome of dental caries as much as saliva does.

Dental caries is a multifactorial disease and the hallmark of this is demineralization which is initiated by acidogenic plaque flora and low salivary flow leading to slow clearance, poor buffering, and reduced supply of calcium to repair the altered dental tissues. The saliva, by constantly bathing the teeth and oral mucosa, functions as cleansing solution, a lubricant, a buffer, and an ion reservoir of calcium and phosphate, which are essential for remineralization of initial carious lesion.^1^

Low salivary buffering capacity, low calcium and phosphate levels show a pronounced link to increased caries. Maintenance of the equilibrium between demin-eralization and remineralization depends on the ionic concentration of calcium and phosphate in saliva, which in turn is influenced by AP levels. Variations in the levels of salivary enzymes like AP cause changes in phosphate levels, which lead to initiation and progression of dental caries.^2^

Salivary constituents are affected in various local and systemic conditions. Among the various systemic diseases, DM is the fifth most common metabolic disorder routinely encountered in the world and its prevalence is constantly on the rise.^3^ According to the 6th edition of the International Diabetes Federation, diabetes atlas, India has 3 new cases of type I DM per 1 lakh children of 0 to 14 years. It is a complex multisystem disorder characterized by a relative or absolute insufficiency of insulin secretion and/or concomitant resistance to the metabolic action of insulin on target tissues. There is evidence that diabetic patients have saliva secretion and composition different from nondiabetic subjects. It has been suggested that hyperglycemia is associated with decreased salivary secretion and high salivary glucose levels, particularly in cases of severe insulin deficiency. Consequently, an increased cariogenic challenge in such individuals can be expected.^4^

Alterations in salivary amount and quality affect oral health. The alterations observed may be related to the levels of glucose, calcium, enzyme activity, and IgA as well. Normal glucose level in saliva is 0.5 to 1.00 mg/100 mL, which does not significantly affect oral health or support the growth of microorganisms. However, higher salivary glucose levels favor the proliferation of microorganisms and enhance their colonization on teeth and oral mucous membrane.^5^ Furthermore, it is possible that increased concentration of IgA in saliva have protective role against caries development.^6^

The salivary components in diabetic children too may suffer variations that can be detected by chemical determinations. Despite all the aforementioned risk factors for dental caries, the relationship between dental caries and diabetes remains controversial.^6^

However, most of the clinical trials estimating the salivary parameters are carried out in adults, which dictates the need to recognize significant association between the levels of salivary factors linked to dental caries in children with and without DM. Hence, the present study was aimed to estimate the levels of salivary glucose, calcium, phosphorus, AP, and IgA among diabetic and healthy nondiabetic children with caries and also to analyze the possible association between the caries status and possible caries determinants.

**Fig. 1:**
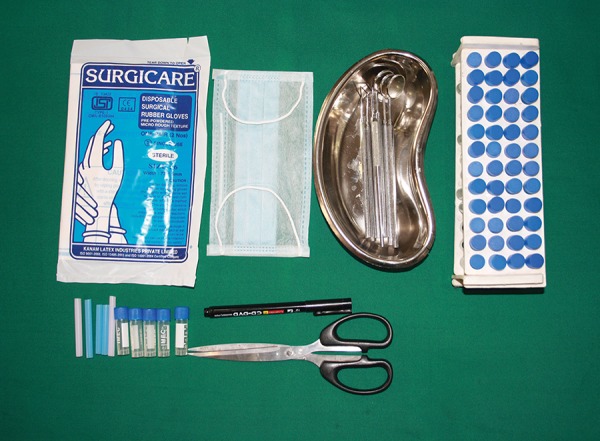
Armamentarium used for the study

**Fig. 2:**
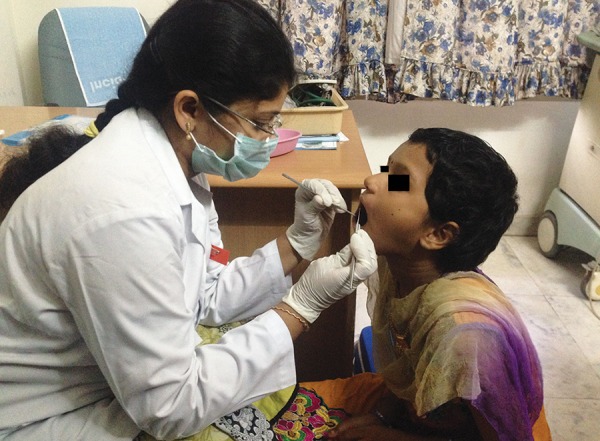
Examination of participants

## MATERIALS AND METHODS

This randomized clinical trial of diabetic and healthy children was conducted after obtaining clearance from the institutional ethical board. The study population was selected based on the duration of DM and hemoglobin A1c levels. To unify the cases, children affected with diabetes with a minimum duration of 2 years and also on medication for the same were selected.

The study comprised a total of 70 children inclusive of both sexes in the age group of 6 to 13 years. Both diabetic and healthy children who had minimal dental caries (DMFT/dmft >1 and <5) were selected and grouped as follows:

- Group I: 35 children with type I DM and on medication for diabetes (study group).
- Group II: 35 healthy children (control group).

### Exclusion Criteria (Study Group)

- Cases with acute oral afflictions, acute systemic infections, and chronic debilitating diseases.
- Patients with severe diabetic complications and other systemic illness or on medications other than those for diabetes.

### Selection of Individuals

Informed consent was obtained from all the children/ parents for participation in the study.

The intraoral examination was performed to assess the DMFT/dmft in both normal and diabetic children (Figs 1 and 2). To unify the cases, children with minimal caries, i.e., DMFT > 1 and < 5, only were included in the study. Children who fulfilled the inclusion criteria were assigned for saliva collection.

The passive drool method was employed for the collection of whole saliva into a small vial.

### Participant Preparation

**Fig. 3:**
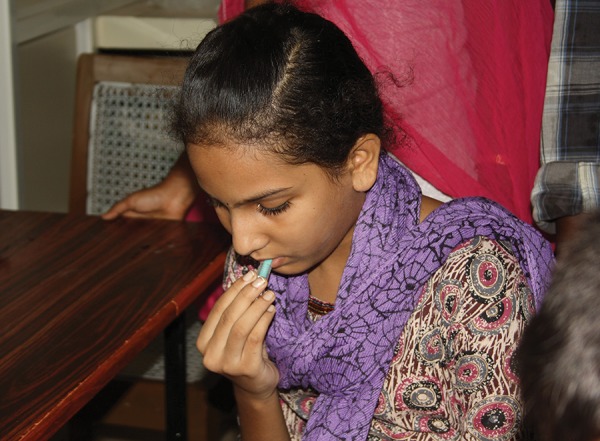
Saliva collection (passive drool method)

**Fig. 4:**
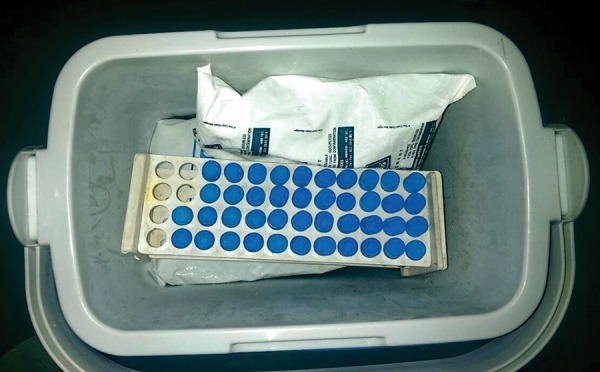
Portable icebox for saliva storage

Participants were instructed not to consume food (neither solids/liquids) 2 hours before and were asked to rinse their mouth with water 20 minutes prior to collection. The circadian rhythm can change the composition of saliva in the same individual at different times of the same day. To control the circadian variation, samples from all the children were collected between 10 am and 11.30 am.^7^ Each research participant was given a straw piece and a cryovial. They were instructed to allow saliva to pool in the mouth and with head tilted forward, saliva was made to drool down the straw and collect in the cryovial (Fig. 3) (It is normal for saliva to foam, so a vial with twice the capacity of the desired sample volume was used.) It was repeated as often as necessary until 2 mL was sufficiently collected. The samples were stored after collection at (4°C) and were transported to the lab in a portable icebox (Fig. 4) within 24 hours.

**Fig. 5:**
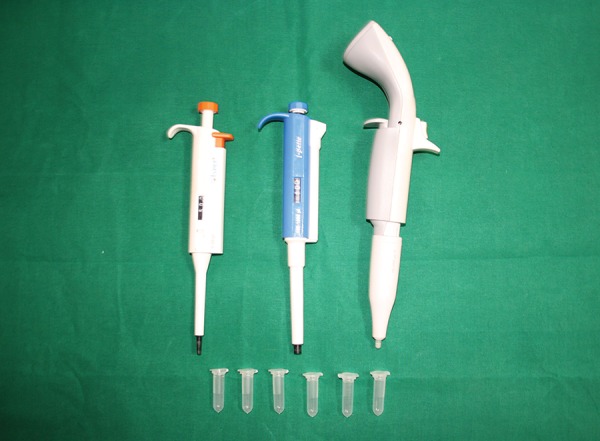
Micropipettes for dilution of saliva

On the day samples were to be assayed, it was brought to room temperature, vortexed, and then centrifuged for 15 minutes at approximately 3,000 RPM (1,500 × g). Assays were performed using only clear saliva, avoiding any sediment present in the bottom of the tube (Fig. 5).

**Fig. 6:**
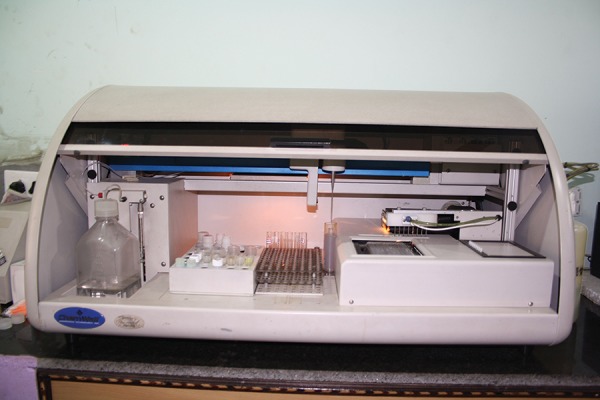
Chemwell autoanalyzer (Awareness tech, USA)

**Fig. 7:**
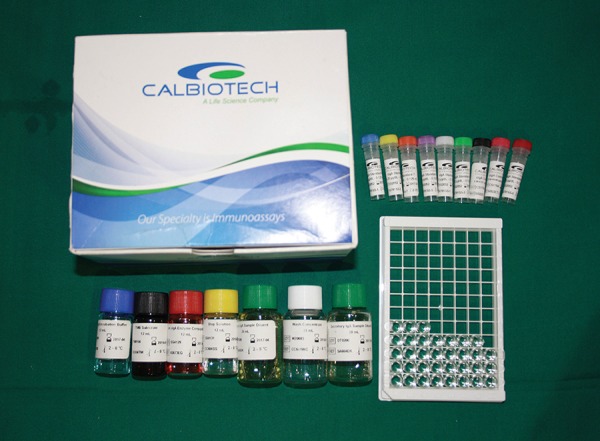
Calbiotech ELISA kit

All the samples were subjected to analyses in a fully automated analyzer (Fig. 6) with biochemistry and ELISA reader (Chemwel plus, CPC diagnostics, Awareness technologies, USA), which works on the principle of atomic absorption spectrophotometer (Fig. 7).

**Fig. 8:**
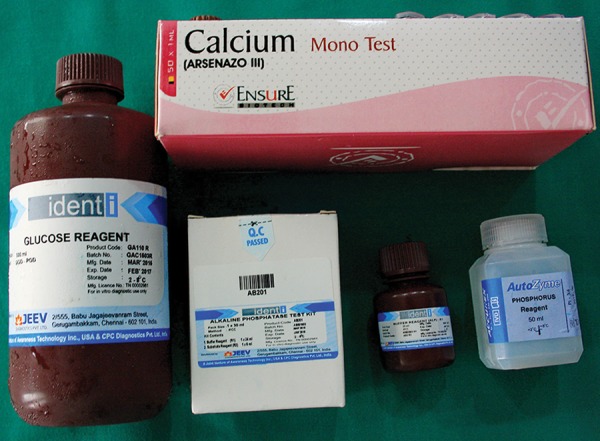
Reagents used for the study

- Salivary glucose estimation was performed using the glucose oxidase-peroxidase end-point method.
- Salivary calcium and phosphorus levels were estimated by colorimetric method (Fig. 8).
- For the estimation of AP, enzymatic assay kit was used that measures the concentration of AP using a direct, plate-based, colorimetric reaction.
- For the quantitative determination of s-IgA, immuno-enzymatic colorimetric method was used. This s-IgA enzyme-linked immunosorbent assay (ELISA) test was based on simultaneous binding of human IgA to two antibodies, one monoclonal immobilized on a microwell plate, the other with polyclonal conjugated with horseradish peroxidase. The s-IgA concentration in the sample was calculated based on a series of standard. The color intensity was proportional to the s-IgA concentration the sample.

**Table 1:** **Table 1:** Mean and SD of diabetic and nondiabetic children with respect to age and gender

*Sample distribution*							
				*Study*		*Control*	
No of subjects				35		35	
Age (yrs)		Mean ± SD		10.1 ± 2.3		8.2 ± 1.1	
		Range		6-13		6-13	
Gender		Male		13		21	
		Female		22		14	

**Table 2:** **Table 2:** Mean and SD of diabetic and nondiabetic children with respect to salivary glucose

*Glucose*									
*Groups*		*Mean*		*SD*		*Min*		*Max*	
Study		4.51		2.83		1.00		12.00	
Control		1.32		1.17		0.00		5.00	
Mean difference		3.19							
t-value		6.09							
p-value		<0.001, HS							

HS: Highly significant

**Table 3:** **Table 3:** Mean and SD of diabetic and nondiabetic children with respect to salivary calcium

*Calcium*									
*Groups*		*Mean*		*SD*		*Min*		*Max*	
Study		2.66		1.75		0.00		8.10	
Control		3.81		3.16		0.00		12.90	
Mean difference		1.15							
t-value		1.91							
p-value		0.06, NS							

NS: Not significant

**Table 4:** **Table 4:** Mean and SD of diabetic and nondiabetic children with respect to salivary phosphorus

*Phosphorus*									
*Groups*		*Mean*		*SD*		*Min*		*Max*	
Study		11.66		2.70		6.70		18.00	
Control		11.77		2.30		1.10		38.20	
Mean difference		0.11							
t-value		0.18							
p-value		0.86, NS							

NS: Not significant

**Table 5:** **Table 5:** Mean and SD of diabetic and nondiabetic children with respect to salivary AP

*AP*									
*Groups*		*Mean*		*SD*		*Min*		*Max*	
Study		22.09		11.07		0.00		48.50	
Control		15.21		7.91		1.10		38.20	
Mean difference		6.89							
t-value		2.98							
p-value		0.004, S							

S: Significant

**Table 6:** **Table 6:** Mean and SD of diabetic and nondiabetic children with respect to s-IgA

*Salivary IgA*									
*Groups*		*Mean*		*SD*		*Min*		*Max*	
Study		36.44		16.30		13.52		72.65	
Control		34.26		12.82		12.39		56.53	
Mean difference		2.18							
t-value		0.65							
p-value		0.52, NS							

NS: Not significant

## RESULTS

Statistical analysis was performed by mean, SD, and Student’s t-test. The range, mean scores, and SDs for diabetic and control group are presented in Table 1. The mean values of salivary components estimated are exhibited in Tables 2 to 6.

In group I (diabetic children), the mean concentrations of salivary glucose, calcium, phosphorus, AP, and s-IgA were 4.51 mg/dL, 2.66 mg/dL, 11.66 mg/dL, 22.09 U/L, and 36.44 mg/dL respectively.

**Graph 1:**
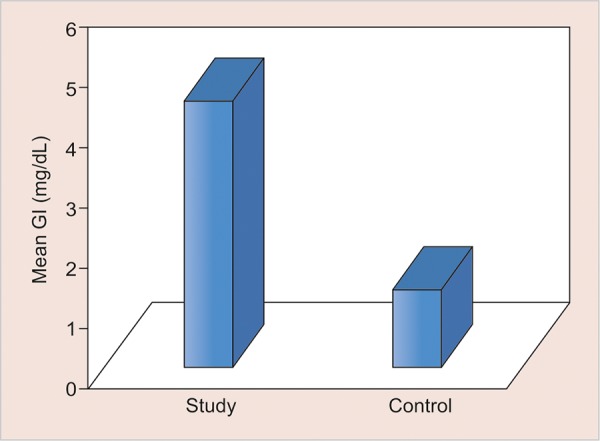
Mean salivary glucose levels in two groups

**Graph 2:**
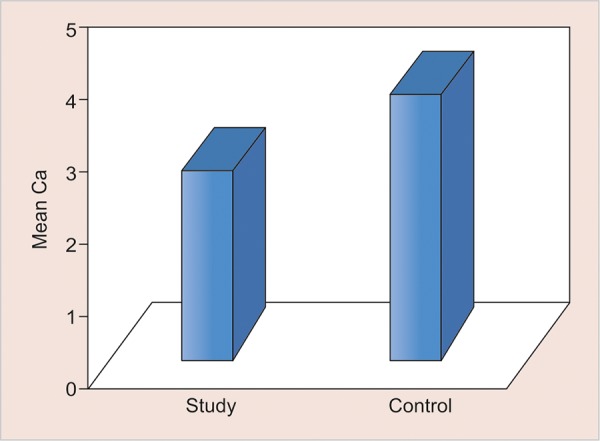
Mean salivary calcium levels in two groups

**Graph 3:**
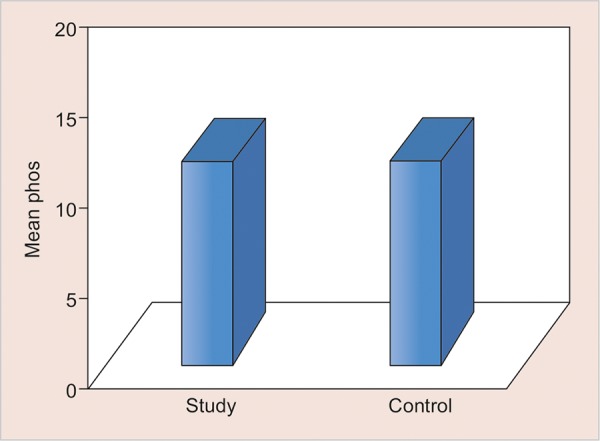
Mean salivary phosphate levels in two groups

**Graph 4:**
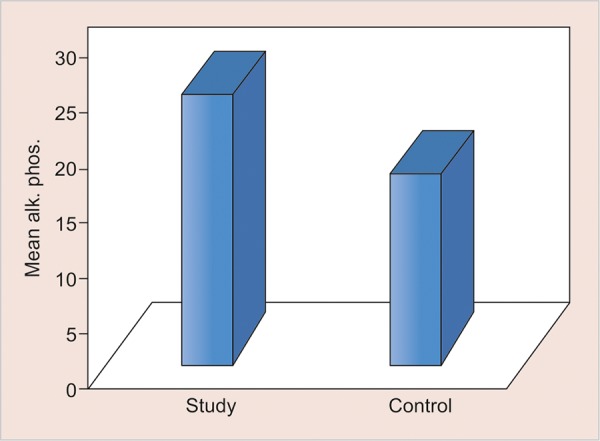
Mean salivary AP levels in two groups

**Graph 5:**
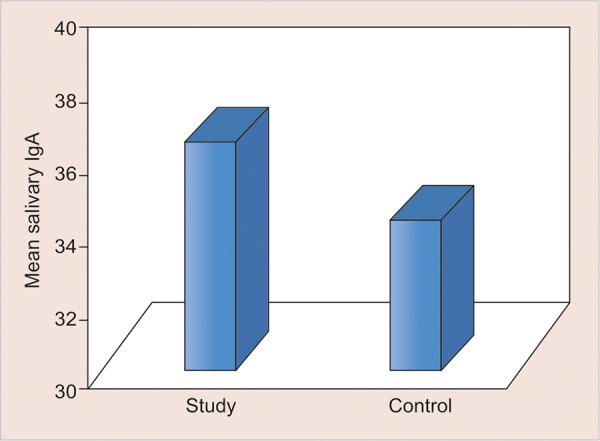
Mean s-IgA levels in two groups

In group II (nondiabetic children), the mean concentrations of salivary glucose, calcium, phosphorus, AP, and s-IgA were 1.32 mg/dL, 3.81 mg/dL, 11.77 mg/dL, 15.21 U/L, and 34.26 mg/dL respectively.

The values of comparison are depicted in Graphs 1 to 5.

## DISCUSSION

One of the goals in dentistry today is the provision of regular care to high-risk groups of the population, among which are the medically compromised children, such as those suffering with type I DM. Continuous monitoring of oral health of these children leads to dramatic improvement of oral ecology and a decrease in the caries incidence.

The present study assessed the various salivary components like glucose, calcium, phosphorus, AP, and s-IgA among diabetic children because these elements have a role in maintaining the equilibrium between the dissolution of enamel by acids produced by oral microbes and saliva of the oral cavity. The study was anticipated to estimate the probable association linked to salivary factors and the risk of systemic disease on child’s oral health when compared with healthy children.

The present investigation included children in the age group of 6 to 13 years, as this is the developmental period of transitional dentition and the duration of overt diabetes may exert an influence on the mineralization of newly erupted teeth and also affect the inorganic constituents of saliva.

The results of the present study indicated significantly higher glucose levels among diabetics when compared with healthy children. These results are in agreement with the reports of previous studies by Panchbai et al^5^ and Abhikshyeet et al.^9^ Earlier reports have quoted various reasons for the elevated glucose content in the salivary secretion of diabetic individuals. Lopez et al stated that the salivary glands act as filters of blood glucose that would be altered by hormonal or neural regulation. Glucose also makes its way from the gingival crevicular fluid into the saliva. Even under pathologic conditions like infections and inflammations of salivary glands, raised glucose levels can be seen.^8^ Persistent hyperglyce-mia leads to microvascular changes in the blood vessels, as well as basement membrane alteration in the salivary glands. This leads to increased leakage of glucose from the ductal cells of salivary gland, thereby increasing the glucose content in saliva.^9^

Decreased secretion of saliva combined with an increased amount of glucose in the saliva and exudate from gingival crevices can presumably be a caries-promoting factor.^10^

Increased levels of glucose in the saliva stimulate bacterial growth and the production of lactic acid, leading to a decrease of the pH and buffering capacity of the saliva, and converts them to risk factors for the development of dental caries. Increased oral acidity resulting from the high level of glucose in the saliva causes a change in the dental biofilm and facilitates the colonization of *Streptococcus mutans* and *Lactobacillus*^11^

Though numerous studies found higher salivary glucose levels in diabetic patients than in nondiabetics, Belazi et al,^11^ Bakianian Vaziri et al,^12^ Gheena et al,^13^ Shahbaz et al,^14^ and Kakoie et al^15^ did not report any difference with respect to salivary glucose levels in normal and diabetic individuals. The difference may be due to the diabetic status and better glycemic control of the studied sample. These differences in the analysis of results might also be due to different methods employed for the measurement of salivary glucose or even the method of collecting the samples. In addition, the level of maintaining oral hygiene and plaque on teeth can influence the salivary glucose levels.

According to Twetman et al,^16^ the cut-off value of stimulated resting salivary glucose concentration for poor metabolic control and caries-associated risk factor is >12.5 mg/L. However, the present investigation showed the mean glucose levels in diabetics to be well within the range, and hence, the associated risk might also be minimized.

### Salivary Calcium and Phosphorus

In the present study, the mean calcium concentration in diabetic children with dental caries was 2.66 ± 1.75 and that of nondiabetic children was 3.81 ± 3.16, which is slightly high. Mean salivary phosphorus levels in diabetic children was found to be 11.66 ± 2.70 and that of nondiabetic children was 11.77 ± 2.30 with a mean difference of 0.11 mg/dL, which is not statistically significant (p > 0.05). Hence, a nonsignificant difference was revealed between calcium and phosphorus levels among the diabetics and healthy children.

As reported by Damle et al,^17^ the levels of calcium, phosphorus, and AP are not significantly altered in caries-active and caries-free persons. Lasisi and Fasanmade^18^ reported that calcium levels in diabetics are not significantly different from that of the nondiabetics. Individuals with increasing DMFT show a decrease in minerals like calcium and phosphorus in both serum and saliva.^19^

The adequate level of calcium, phosphate, and even fluoride is responsible for the significant deposition of these minerals in plaque that greatly reduces the development of caries in the adjacent enamel. This indicates that diabetic patients are more prone to develop dental caries if the calcium, phosphorus level decreases and the levels of AP increase.^20^ Since the values estimated among diabetics in the present study were on par with the healthy children with respect to salivary calcium and phosphorus, the susceptibility also may be considered to be minimum.

Factors to be taken into consideration here include newly diagnosed cases if any, among the sample population studied because diabetes of less than 2-year duration may hardly have any influence on dental development.^21,22^

### Alkaline Phosphatase

Alkaline phosphatase is a nonspecific phosphomonoester-ase that has different isoenzymes produced by different cell types, such as polymorphonuclear leukocytes, osteoblasts, macrophages, and fibroblast within alveolar bone and/or salivary glands. It has been shown in different studies that higher AP activity is related to periodontal disease and dental caries and it seems that the function of this protein is relatively dependent on salivary pH and buffering.^23^

Mean salivary AP levels in diabetic children (22.09 ± 11.07) were much higher when compared with nondiabetic children (15.21 ± 7.91) with a mean difference of 6.89 mg/dL. A statistically significant difference (p < 0.05) was observed in the present investigation between the two groups with respect to salivary AP levels.

Hegde et al,^22^ who reported AP activity in saliva to be higher in diabetic adults, substantiate the findings with the results of the present study. It is well established that initial caries lesion formation seen clinically as the white spot lesion is marked by a subsurface demineralization with an intact surface layer which can be remineralized, reestablishing the demineralization-remineralization equilibrium.

Studies documented by Vijayaprasad et al^24^ and Gandhy and Damle^25^ have shown a positive and direct correlation between salivary AP level and dental caries. The same authors in a different study reported that there was an increase in the levels of caries with an increase in the levels of AP levels in both serum and saliva. They reported calcium levels in serum and saliva to be decreased in caries-prone individuals, thus showing a significant correlation between serum and salivary AP and calcium levels.

Vijayaprasad et al^24^ and Bakas^20^ also reported AP activity to be significantly higher in caries-prone groups, but observed no significant difference of calcium and phosphorus levels between caries-free, minimal caries, and caries-prone groups. The present study too did not reveal any significant difference between calcium and phosphorus levels between type I DM and nondiabetics. Although salivary AP can balance demineralization and remineralization processes of enamel, there is no evidence regarding its effects on the concentrations of calcium and phosphate in saliva.

In contrast to the present study, Jazaeri et al^23^ reported no significant relation between salivary AP activity and calcium and phosphate concentrations in saliva. It seemed that the other controlling mechanism was responsible for maintaining calcium and phosphate at a normal range.

### Salivary IgA

Mean s-IgA level in diabetic children was 36.44 ± 16.30 and that of nondiabetic children was 34.26 ± 12.82. Though the levels of s-IgA were high among diabetic children, the difference was not statistically significant (p > 0.05).

Salivary IgA antibodies help oral immunity by preventing microbial adherence, neutralizing enzymes, toxins, and viruses, or by acting in synergy with other factors, such as lysozyme and lactoferrin. Gandhy and Damle^25^ reported that s-IgA levels in children with no caries were significantly higher, suggesting a possible protective role of IgA in the prevention of dental caries. It could be due to the deficient transport mechanism, stimulation of the immune system via pulp, deficient local immunoglobulin synthesis, and molecular size of IgA.

In the present study, on comparison of s-IgA levels, diabetic children had higher levels of s-IgA when compared with nondiabetic children. However, the difference was not statistically significant. This is in agreement with the results of Rashkova et al^26^ and Bakianian Vaziri et al^12^

who reported that IgA levels are not significantly altered in either type I DM or type II DM compared with healthy controls. Contrary to this, Harrison showed significantly higher levels of s-IgA in uncontrolled diabetic children only, whereas no difference between controlled DM and healthy children.^27^

Watanabe et al^28^ demonstrated a significant increase in s-IgA levels in diabetic patients. They suggested that it could be related to local factors, such as calculus and higher bacterial plaque accumulation in these patients. The findings of this study on s-IgA levels were in contrast with other studies; these differences may be due to differences in the type of saliva collected (stimulated or unstimulated), the saliva collection methods, the stage and the metabolic control status of the disease.

The average values of s-IgA in the saliva of children with diabetes are close to those of the healthy children in the present study. Due to the initial phase of diabetes development, the endocrine system does not seem to influence the local secretary oral immunity to a greater extent.

The present study showed nonsignificant values with respect to calcium and phosphorus. This is attributed to the fact that the systemic effects of diabetes might not have much influence on tooth mineralization and concentration of inorganic constituents of saliva. However, higher glucose levels among diabetics may be due to more permeability of salivary glucose. Levels of s-IgA too were not a significant factor among diabetics and hence protection against developing caries can be anticipated on par with the normal children.

## CLINICAL SIGNIFICANCE

The salivary factors evaluated here may prove to be useful measures of caries determinants in diabetic children which may help pediatric dentists to combat the ill effects of diabetes on oral health and target preventive measures appropriately.

## CONCLUSION

The following conclusions were drawn from the present study:

- Despite a difference in systemic health status, the diabetic children showed minimum variation in salivary composition and oral health.
- There was no correlation in the levels of salivary calcium, phosphorus, and s-IgA levels among diabetic and healthy children.
- An elevation in the levels of salivary glucose and AP was evident in diabetic children, which can be risk markers for dental caries.

However, the clinical interpretation of results obtained in this case-control study should be further substantiated incorporating a larger sample size in order to conclude the specific role played by saliva as a host-protective factor in diabetic children.
